# Aluminum—The Metal of the Future

**Published:** 1892-01

**Authors:** 


### EDITORIAL.

### Aluminum—The Metal of the Future.

An article in the January number of the *Cosmopolitan* makes some very important and interesting statements in regard to the method of procuring and working aluminum; its properties and uses. The method of separating it from the elements in nature with which it is associated has been, until within a recent period, a very difficult and slow process. Great progress in this respect has been made, however, within the last half dozen years. By the older methods of production, aluminum cost more than its weight in gold. The valuable properties of the metal have been so fully recognized for the last half century and more that there have been persistent and continued experimentations carried forward with a view of cheapening the process until now it is produced for fifty cents per pound, which, comparing its bulk with iron would not be more than ten or twelve cents per equal pound volume, and the strong probabilities are that its production will be still farther cheapened; to what extent, however, we will not venture to predict.

It is an important fact in connection with this subject that next to oxygen and silicon aluminum is the most abundant element on the earth's surface; it is found every where; does not require to be mined from the depths of the earth, but is all over its surface. A great and radical step was made in separating this metal in 1886 by Mr. Chas. M. Hall, of Oberlin, O., who applied for a patent covering the decomposition by electricity of a fluid bath, consisting of aluminum fluoride and sodium fluoride in which alumina had been dissolved, and though this was contested in the Patent Office, Mr. Hall was allowed priority of invention and the patents were granted him. The process is now installed on a large scale at Kensington, on the Alleghany River, sbout eighteen miles above Pittsburgh, where with dynamos of 500 horse power, about 500 pounds of aluminum are produced daily. The process is as simple in operation as in theory. The baths are kept melted by the heat of the immense electric current passing through them, which at the same time decomposes the alumina in solution without decomposing the bath in which the alumina is dissolved. * * *.

"In Mr. Hall's process the electrolytic preparation of aluminum has reached its climax of simplicity, and when fully developed, of economy. By this process aluminum is now being manufactured at a cost less than fifty cents per pound. The present practical application of this process is in a large measure due to the energy and ability of Cap. A. E. Hunt, of Pittsburgh, President of the company.

"The present status of the aluminum industry is as follows: There are six establishments actually producing pure aluminum, viz.: The Pittsburgh Reduction Co., working Hall's patents; The Metal Reduction Co., in Lancashire, England, working Hall's English patents; The Aluminum Actien-Gesellschaft, working the Heroult process at the Rhine Falls; The Cowles Electric Smelting and Aluminum Co., at Lockport, N. Y., The Cowles Syndicate Co., at Stoke-on-Trent, England, and the Works of Bernard Bros., at Criel, France, who are using an electrolytic process somewhat intermediate in its nature between Deville's electrolytic process and Hall's."

Questions as to the utility and purposes for which aluminum may be used are frequently asked. At the present stage of its development, in this respect, it is impossible to speak with any degree of fullness and definiteness. It has already been used in many directions; it possesses various valuable properties; its weight being so much less than that of any other known metal, and especially of the metals most used in the arts, is a very important quality indeed. In the words of the writer above referred to, aluminum is only $2\frac{1}{2}$ times as heavy as water, while iron is $7\frac{1}{2}$, brass 8 times, copper 9 times, silver $10\frac{1}{2}$, lead 11, gold 19 and platinum $21\frac{1}{2}$ times.

Many uses have been suggested to which aluminum may be applied, but while considering this subject it must be borne in mind that aluminum while only one-third as heavy as steel, is also only one-third as strong; still a rod of aluminum of the same weight as a rod of steel would have the same strength; for use in large and heavy structures such as bridges, buildings, etc., this fact should be borne in mind. But aluminum alloyed with other metals gives another aspect to this question; some of these are well nigh, if not altogether, the strongest metals we have: five or ten per cent. of aluminum added to copper forms a beautiful bronze of a golden color and is as strong as ordinary steel; a small portion of copper or titanium added to aluminum makes it much stronger without perceptibly increasing its weight.

Another property of aluminum that adds to its value is its resistance to many strong corrosive agents; it is less easily acted upon by the ordinary corrosive agents than silver; it resists in a very marked degree sulphurous vapors and fluids, these having not the slightest blackening effect on it, while silver is rapidly corroded by such agents; this property gives aluminum a decided advantage over silver for all sorts of table ware, and for ornamental work. The acids of the body have no effect on aluminum, so that, surgeons use all sorts of instruments made of it with great satisfaction, as to the cleanliness, as well as using it for suture wire, supports, tubes, etc. Nitric acid has little or no effect upon it.

One of the most important uses of aluminum is for cooking utensils; three important properties make it of value, viz.: lightness, resistance to corrosive action, and great conductibility of heat; the reduction of weight in such vessels is an important matter to those who handle them; the second property enables them to last much longer than those made of the ordinary materials; and by the third it is proposed that better cooking can be done. Aluminum has not only the property of readily conducting heat, but it also has a remarkable power of retaining it. Almost daily some new uses for aluminum are suggested, and actual application made; these are greatly increasing since the great reduction in price.

One of the chief difficulties in the way of working aluminum has heretofore been the difficulty of soldering it, but recently this problem has been completely solved by Mr. Joseph Richards, of Philadelphia. Aluminum can now be soldered as easily and as firmly as copper or sheet iron.

In regard to aluminum for dental purposes, it has been used with varying success in some degree for over twenty years.

It has been used by casting and in plates. James B. Bean, D.D.S., of Baltimore, Md., was the first who formed dental plates by casting. It was, however, by a very difficult and uncertain process, and at that time it was almost impossible to secure aluminum free from baser metals which very greatly impaired its permanency in the mouth.

In later years the process of casting has been very much simplified by Dr. Carroll, of New York City, and the success greater from the fact that pure aluminum is now easily obtained; but at the best, this is a process requiring considerable experience, practice and great care. Aluminum for artificial dentures is more easily worked in the form of plate, just as gold or platinum, and as easily manipulated as either of these metals; teeth can be attached to it either by soldering, or on some accounts better with rubber, as the sulphur in the rubber does not affect the metal in vulcanizing. This metal is as acceptable to the tissues of the mouth as any metal that is used for dental purposes, and when well adapted to the parts makes one of the most comfortable artificial dentures that is made. Some experiments have been made with alloy of aluminum for dental plates; we are not aware, however, that any thing very satisfactory has been, as yet, obtained in this direction; but we will not pretend to assert that success may not in the near future be attained in this direction.

